# Biomarkers of prognosis and efficacy of treatment in OA

**DOI:** 10.1186/1471-2474-16-S1-S2

**Published:** 2015-12-01

**Authors:** Yves Henrotin

**Affiliations:** 1 Bone and Cartilage Research Unit, Arthropôle Liège, University of Liège, CHU Sart-Tilman, Institute of Pathology, 4020 Liège, Belgium

## 

OA is a disease affecting the metabolism of all joint tissues leading to structural changes visible by imaging techniques. Unfortunately, features visible by imaging are in most cases irreversible and progressively moving towards worsening. One challenge for the next decade will be disease detection at the early stage when the first molecular/metabolic changes appear in joint tissues. Another challenge is to develop tools to assess the efficacy of OA treatment on the natural history of the disease. Therefore, there is an acute need for reliable biological markers that can facilitate earlier diagnosis of OA, predict the progression of the disease and evaluate the efficacy of therapeutic modalities.

A recent literature review resulted in the identification of 16 biochemical markers investigating cartilage matrix turnover. Nine concerned collagen type II degradation (Coll2-1, Coll2-1NO2, CTX-II, Helix-II, C2C, TIINE, CIIM) and synthesis (PIIANP, PIICP). Keratan sulphate, chondroitin sulphate 846 (CS846) and ARGS-aggrecan fragment investigate proteoglycans degradation. Serum cartilage oligomeric matrix protein (COMP), deamidated-COMP (D-COMP), fibuline-3 fragments (Fib3-1 and Fib3-2) were the other biochemical markers that are considered as markers of cartilage matrix metabolism.

Risedronate and strontium ranelate, two drugs currently used to treat osteoporosis decreased urinary CTX-II levels suggesting that they can modulate cartilage metabolism, even if they did not alter radiological progression. However, recently, it was demonstrated that CTX-II was more strongly associated with bone markers (i.e. uNTXI, uCTXI, serum PINP, and osteocalcin) than with other cartilage markers (PIIANP, sCS846, sCOMP), while the “other” cartilage markers were not so strongly associated with the bone markers. These data indicate that CTX-II might reflect bone rather than cartilage metabolism. In an exploratory study investigating the effects of three intra-articular injections of hyaluronic acid (Hylan GF-20) on the evolution of 10 biochemical markers, we have demonstrated that uCTXII, sColl2-1 and sColl2-1NO2 levels were significantly affected by treatment suggesting that these markers are sensitive to metabolic change occurring in one single joint. More recently, we have observed that three months treatment with bio-optimized curcumin significantly decreased sColl2-1 level in 24 patients with knee OA, suggesting that sColl2-1 could be a companion marker to assess curcumin efficacy at an individual level and in the next phases of its clinical development.

Although many OA-related biomarkers are currently available they exist in various states of qualification and validation. At this time, none of the existing biomarker can be considered as a surrogate marker of clinical and imaging feature for the diagnosis or prognosis of the disease. In this context, the recent development of large cohort designed to qualify biomarker will accelerate biochemical marker implementation in clinical research.

